# The Role of Inner Speech in Executive Functioning Tasks: Schizophrenia With Auditory Verbal Hallucinations and Autistic Spectrum Conditions as Case Studies

**DOI:** 10.3389/fpsyg.2020.572035

**Published:** 2020-09-17

**Authors:** Valentina Petrolini, Marta Jorba, Agustín Vicente

**Affiliations:** ^1^Centro de Investigación Micaela Portilla, Department of Linguistics and Basque Studies, University of the Basque Country (UPV/EHU), Vitoria-Gasteiz, Spain; ^2^Ikerbasque, Basque Foundation for Science, Bilbao, Spain

**Keywords:** inner speech, executive functions, autism spectrum conditions, schizophrenia, auditory verbal hallucinations

## Abstract

Several theories propose that one of the core functions of inner speech (IS) is to support subjects in the completion of cognitively effortful tasks, especially those involving executive functions (EF). In this paper we focus on two populations who notoriously encounter difficulties in performing EF tasks, namely, people diagnosed with schizophrenia who experience auditory verbal hallucinations (Sz-AVH) and people with autism spectrum conditions (ASC). We focus on these two populations because they represent two different ways in which IS can fail to help in EF tasks, which can be illustrative for other mental conditions. First, we review the main components of EF (see section “Executive Functions”). Then we explain the functions that IS is taken to perform in the domain of EF (see section “Inner Speech and Executive Functions”) and review the evidence concerning problems about EF in the two populations of our study: Sz-AVH (see section “Executive Functions and Inner Speech in Sz-AVH”) and ASC (see section “Executive Function and Inner Speech in ASC”). After this we further detail our account about what a properly functioning IS can do for both populations and how different IS profiles may impact EF performance: in the case of Sz-AVH, the uncontrolled and intrusive character of IS negatively affects EF performance, whereas in ASC, EF is not sufficiently supported by IS, given the tendency in this population to present a diminished use of IS (see section “IS in ASC and Sz-AVH: How It Relates to EF”). We finally briefly discuss Attention Deficit/Hyperactivity Disorder (ADHD) and Developmental Language Disorders (DLD) (see section “Further Considerations”).

## Introduction

Several theories propose that one of the core functions of inner speech (IS) is to support subjects in the completion of cognitive effortful tasks, especially those involving executive functions (EF) (Vygotsky, 1987; Fernyhough, 1996, 2004; Winsler et al., 2009). This seems to be the case in the majority of the neurotypical population, although the use of IS varies from subject to subject (Heavey and Hurlburt, 2008). Recurring themes in very different questionnaires refer to the use of IS in tasks that require self-control or self-regulation, that is, in processes involving EF. In fact, ever since Vygotsky (1987), many authors have taken it that the main function of IS is related to EF: young children, in a first developmental step, use overt self-talk in tasks such as planning, inhibition, attention-focusing, etc., in ways that mimic patterns of conversations with their caregivers. In the next step, they internalize such self-talk without altering its purpose. Empirical investigation also shows some support for the idea that IS is involved in EF processes, from working memory (Baddeley, 1992) to task-switching (Emerson and Miyake, 2003) or planning (Alderson-Day and Fernyhough, 2015).

In this paper we focus on two populations who notoriously encounter difficulties in completing EF tasks, namely, people with autism spectrum conditions (ASC),^1^ and people diagnosed with schizophrenia who experience auditory verbal hallucinations (Sz-AVH). ASC and schizophrenia have already been compared to one another with respect to shared clinical features such as social withdrawal, communication impairments, or failure to make eye contact (Dvir and Frazier, 2011). Here we aim to compare these two populations along a new dimension, i.e., EF performance and the role that IS plays in it. By studying these two different populations we also try to understand more clearly *what cognitive functions IS supports* and what structure it has to embody to perform them. Given the nature of the IS profile and the EF issues that schizophrenia patients with AVH encounter, it is pertinent that we focus on this subgroup. With respect to ASC, we focus on studies investigating the presence and use of IS in this population. In general, we know about the functions that IS performs mostly through experiments that use verbal interference (the so-called dual-task studies, where the secondary task is specifically designed to disrupt performance on the primary task). Yet, it is not clear to what extent IS is a tool among others (e.g., it can be easily substituted by visual imagery, or even by “unsymbolized thinking”: Hurlburt and Heavey, 2006; Hurlburt, 2011), or whether it has some features that make it special. It is also not clear whether gaining cognitive control depends on IS simply being “present” (just talking to yourself) or on it being present *in a certain way* (IS has to have certain properties or take a certain form). By comparing ASC people and people with Sz-AVH along the dimension of IS, we suggest that these populations may represent two extreme poles with respect to IS profiles. This might in turn help us to understand other IS profiles in different conditions (i.e., Attention Deficit Hyperactivity Disorder, Obsessive-Compulsive Disorder, depression or anxiety).

The plan for the paper is as follows. First, we review the main components of EF to have a clear grasp of the cognitive processes underlying them (see section “Executive Functions”). Then we explain the functions that IS is taken to perform in the domain of EF (see section “Inner Speech and Executive Functions”) and review the evidence concerning problems about EF in the two populations of our study: Sz-AVH (see section “Executive Functions and Inner Speech in Sz-AVH”), and ASC (see section “Executive Function and Inner Speech in ASC”). After this we further detail our account about what a properly functioning IS can do for both populations and how issues with IS may impact EF performance (see section “IS in ASC and Sz-AVH: How It Relates to EF”). More specifically, we suggest that people with Sz-AVH and ASC fail to properly recruit IS in executive tasks, although they do so *in different ways*. On the one hand, people with Sz-AVH would have a hard time controlling and channeling their IS toward the relevant task, to the extent that their performance would be hindered by intrusions or distractors coming from their own IS. On the other hand, typically, people with ASC would rely less on IS when completing EF tasks, often resulting in poorer performance. In relation with ASC, we finally discuss Attention Deficit Hyperactivity Disorder (ADHD) and Developmental Language Disorders (DLD) (see section “Further Considerations”).

## Executive Functions

The notion of EF is often used within psychiatry and clinical psychology as an umbrella term that encompasses a variety of skills and abilities, such as working memory, updating, shifting, inhibition, planning, problem-solving, or reasoning (see Miyake et al., 2000; Diamond, 2013 for a review). There seems to be some consensus that these skills and abilities form part of EF, even though there is no agreement concerning how to precisely characterize EF, except by relating it to other notions such as self-regulation, self-management or control. These abilities are routinely measured in experimental and clinical settings through a number of tests and measures, e.g., the Tower of London test, the Wisconsin Card Sorting Test (WCST), the Stroop test, etc. However, experimental research on EF has been said to suffer from the so-called “task impurity problem” (Snyder et al., 2015), which appears when the administration of a certain task prompts the use of several different cognitive processes within the whole construct, thus leading to potentially conflated results regarding *specific* EFs (e.g., a subject’s score on the WCST might depend on the recruitment of task-switching, planning, and working memory: Geurts et al., 2009).^2^

The different components of EF are related to different areas in the brain (Vivanti et al., 2019) and may even belong to different “systems” or ways of processing information (Friedman and Sterling, 2019). Several researchers distinguish between core EF and higher-order EF as follows:

o **Core EF*: working memory, updating, inhibitory control, task-switching (Miyake et al., 2000; Lehto et al., 2003; Diamond, 2013; Logue and Gould, 2014).*

o **Higher-order EF*: planning, problem solving, reasoning, verbal fluency (Collins and Koechlin, 2012; Lunt et al., 2012; Snyder et al., 2015).*

As it can be seen, these two groups of cognitive abilities are obviously related to self-regulation or self-control, but they seem to pertain to different cognitive realms. *Core EF* skills relate to basic-level processes that arise relatively early in development and are evolutionarily ancient. We briefly review them below.

*Inhibition* refers to the ability to suppress irrelevant, interfering information and impulses, or contextually inappropriate responses, including emotional reactions. In fact, inhibition encompasses a range of processes as different as selective attention, cognitive, emotional, and behavioral or motoric inhibition (Diamond, 2013). All the habitual tasks designed to track this construct, such as Go/No-Go tasks, the Stroop test, the Day/Night Task, or the Knock/Tap task (Joseph et al., 2005), measure the ability to suppress preponderant responses.

*Working memory* (WM) is the process through which information is actively held in mind for very short periods of time while performing a task (Baddeley, 1992). It also involves monitoring, coding, and appropriately revising relevant information, with the latter at times known as *updating* (Miyake et al., 2000). Habitual measures of WM are the keep track task, the tone monitoring task, and the letter memory task. The Corsi Block test is also often used for this purpose, as it requires subjects to keep a specific sequence in mind, i.e., to touch blocks in the same order as shown by the experimenter. Let us note that *updating* seems to be an independent skill: indeed, subjects or systems could be good at keeping information ready for use as well as at manipulating it, while at the same time being utterly repetitive in their behavior.

Finally, *task-switching* is the ability to shift to different thoughts or actions depending on situational demands (Geurts et al., 2009). It may be characterized as the ability to disengage from an irrelevant task and engage in a relevant one. Task-switching is also often referred to as “cognitive flexibility” (CF), which is usually construed as the opposite of rigidity. Issues with flexibility result in perseveration errors or the repetition of the same response despite varying stimulus (Miyake et al., 2000). However, according to some, CF builds on WM and inhibition as it requires us “to inhibit (or deactivate) our previous perspective and load into WM (or activate) a different perspective” (Diamond, 2013, p. 14). Flexibility thus construed would therefore work as an umbrella term encompassing Core EF, i.e., a combination of WM, inhibition, updating and shifting. Common examples of CF measures are the Plus-minus task, the Number-letter task (Miyake et al., 2000), the Wisconsin Card Sorting Task, the Unusual Uses Task (UUT), and the Intradimensional–Extradimensional Shift task (ID/ED) (see Geurts et al., 2009). Notably, most of these tests involve all the components of Core EF, i.e., WM, inhibition, updating, and switching. Thus, ‘cognitive flexibility’ may simply be another label for ‘Core EF’: to avoid confusion, in what follows we prefer to limit our discussion to task-switching alone, without including CF as a separate component of Core EF.

Higher-order executive functions (HO-EF), on the other hand, relate to cognitive processes and abilities that arise later in development, involve concepts, can be explicitly taught, and appear in fewer species. Some authors link these abilities to System-2 (i.e., conscious, serial, and effortful) processing (Friedman and Sterling, 2019). HO-EF routinely include reasoning and planning. *Reasoning* is a broad notion that applies to a range of different processes such as problem solving and decision making, as well as to seeing patterns or relations among items, or figuring out abstract concepts underlying analogies (Diamond, 2013). As such, it encompasses, although it does not fully correspond to, the notion of “fluid intelligence.” Some of the most common reasoning measures are Raven Matrices, or averaging performance on a diverse battery of tasks (e.g., Hotel Task, Iowa Gambling Task, etc.) – see Roca et al. (2014). *Planning*, in turn, involves choosing and implementing a strategy in routine or new situations in which a sequence of actions must be monitored, judged and updated in light of a pre-specified goal (Hill, 2004; Ward and Morris, 2004). Habitual measures of planning include the Tower of London, the Tower of Hanoi, or Mazes.

As will become clearer in the remainder of the paper, the distinction between Core EF, HO-EF, and their different sub-components proves helpful in acquiring a better understanding of populations who experience difficulties completing executive tasks. We are interested in uncovering more specific ways to characterize the challenges and successes that people with ASC and Sz-AVH experience in the different domains of EF. For instance, it makes a significant difference (both experimentally and conceptually) to understand someone’s performance in an EF task in terms of using resources differently, or inefficiently, or failing to recruit them altogether. Focusing more specifically on different EF components also allows us to offer a more precise explanation of the difficulties encountered by ASC and Sz-AVH populations, one that hinges on their use of IS. EF tasks appear prominent within the research on mental and developmental conditions, such as ASC and schizophrenia (see Snyder et al., 2015 for a review). Although a number of studies reveal that subjects affected by either of these conditions tend to perform poorly in EF tasks (Russell et al., 1999; Ibanez-Casas et al., 2013; Craig et al., 2016), the reasons behind these results are yet to be fully understood.

## Inner Speech and Executive Functions

After James (1890), IS is usually characterized as “the little voice in the head.” It can be seen as an action (subjects perform speech acts with it) and as auditory imagery (if the focus is on the experiential part). IS seems to result from an aborted act of (overt) speech: subjects put a message into words, using the syntax, semantics, and phonology of their language but aborting the motor commands right after they are issued (Lśvenbruck et al., 2018). IS is used for many purposes, from avoiding boredom to reflecting about oneself, but, since Vygotsky (1987), many authors take it that the most fundamental function of IS is self-regulation (for a critical review of this notion, see Langland-Hassan and Vicente, 2018). Indeed, many have made the connection between EF and IS. With respect to *working memory*, IS has been considered part of the phonological loop (Baddeley and Hitch, 1974; Baddeley, 1992), namely a system that, together with the visuospatial sketchpad, supports the central executive in a number of activities such as storing, manipulating, and coordinating information (Mulvihill et al., 2020). Regarding *inhibition*, IS is said to be used for self-control purposes as it *enhances* the ability to restrain our impulses (see Tullett and Inzlicht, 2010 for a study with adults), although it doesn’t appear as necessary for inhibiting responses. Similarly, IS is not key to *task switching* habitual tasks (e.g., in the WCST) but *enhances* performance in these cases as well (see Zelazo, 2004; Cragg and Nation, 2010 for evidence about children). In particular, it has been stressed that IS is not directly responsible for the process of switching between tasks but rather has a more circumscribed role. The studies conducted by Emerson and Miyake (2003) with adults underscore an important self-cueing function played by IS, especially in circumstances where external cues are limited and the subject has to rely on additional self-direction. Such self-cueing would take the form of task-relevant sub-vocalizations, such as “add,” “subtract,” etc. (p. 162). Notably, in these cases IS seems to play a role in making some task-relevant mental states conscious.

With respect to HO-EF like *planning*, IS is not a necessary tool in experimental task performance, at least in adults (Alderson-Day and Fernyhough, 2015), although it has a role again in supporting it. Williams et al. (2012) used a dual task paradigm and showed that neurotypicals performed worse without the aid of IS in a Tower of London task. However, verbal interference did not affect adults with ASC, as we will see, which apparently means that IS may enhance planning performance only in those subjects who regularly use IS for these and possibly other functions. However, it must also be noted that although towers can be planned verbally, they are fundamentally a visuospatial task, so it is unclear whether using IS in other kinds of planning may be actually enhancing performance or even necessary across the board.

As for the last HO-EF, *reasoning*, IS does not appear necessary for logical reasoning and problem solving either (Alderson-Day and Fernyhough, 2015). Actually, some kinds of logical reasoning are present even in prelinguistic children (Cesana-Arlotti et al., 2018). However, both planning and reasoning as typically conceived (i.e., as System-2 effortful, serial and conscious processes), do routinely recruit IS, at least in the neurotypical population. According to some authors, in fact, IS is essential to conscious thinking in general (see Jackendoff, 1996; Bermudez, 2003), and according to other researchers, forms of reasoning involving abstract concepts might be highly facilitated by the use of IS, given that representation of such concepts involves inner articulation of the words associated with them (Borghi et al., 2018).^3^

In general we can summarize the results of the empirical research on the use of IS in EF tasks as being a core feature in (verbal) WM, and mainly enhancing performance in inhibition and task-switching, as well as in planning and reasoning. However, the view that emerges is clearly constrained by the tasks employed to measure the abilities in question.

## Executive Functions and Inner Speech in Sz-AVH

Schizophrenia is a heterogeneous mental condition characterized by a range of cognitive, behavioral, and emotional manifestations. Its description within the DSM-5 distinguishes between positive symptoms (e.g., delusions and hallucinations) and negative symptoms (e.g., diminished emotional expression). Auditory verbal hallucinations represent one of the hallmarks of schizophrenia (Henriksen et al., 2015), although there is evidence that these experiences are quite frequent in non-clinical populations too (see Allen et al., 2006; Johns et al., 2014). AVH are often characterized as “the most typical single symptom in schizophrenia” (Wing et al., 1974; David, 1999), being experienced by 60–80% of people diagnosed with such condition (Sartorius et al., 1986), and they are considered a “marker” of a psychotic episode (Hugdahl et al., 2009).^4^ Despite the complexity surrounding AVH manifestations, in this paper we focus on AVH within the context of schizophrenia (Sz-AVH), and on the IS profile exhibited by this subgroup in relation with EF.

Before narrowing the focus to the Sz-AVH subgroup, we review some evidence regarding EF issues in schizophrenia. Common measures of *inhibition* and *shifting*, such as the Stroop test and the WCST, reveal the difficulties that people with schizophrenia exhibit with “changing attention from one aspect of the stimulus to another” (Ibanez-Casas et al., 2013, p. 6). Similarly, they appear to perform poorly in *updating* and in *planning* tasks like the Tower of London (Grover et al., 2011). The meta-analysis recently conducted by Snyder et al. (2015) reveals that “the largest EF deficits are found for individuals with schizophrenia, with large effect sizes on measures of shifting, inhibition, updating, visuospatial WM, and verbal manipulation, and a medium effect size for simple verbal WM maintenance” (p. 10). Compromised cognitive functioning thus appears to be a pervasive feature of schizophrenia, with 70–80% of patients exhibiting cognitive deficits in the form of severe general intellectual impairment and EF difficulties in planning, switching, updating, and working memory (Reichenberg, 2010; see also Wobrock et al., 2009).

Subjects affected by schizophrenia appear to fare particularly poorly in tasks measuring *inhibition*, both in terms of response (i.e., self-control and impulsive acting) and interference control (i.e., cognitive inhibition; see Diamond, 2013 on this distinction). More evidence on this point comes from the so-called Jumping-To-Conclusions tasks (JTC), where participants are asked to rate the likelihood of a future event after observing only a few instances. One common JTC measure is the so-called beads task, in which participants are shown two jars containing beads of different colors (e.g., pink and green): one jar will typically have significantly more pink than green beads and the other will have significantly more green than pink beads. The jars are then hidden and participants are shown a sequence of beads apparently being drawn from one of the two jars. After each draw, participants are asked if they are ready to make a decision about which jar the beads were being drawn from, or if they would like to continue with the draw. Many studies report that people with schizophrenia are prone to jumping to conclusions on the basis of fewer elements, i.e., they make a decision about which jar the beads are being drawn from on the basis of significantly fewer beads than controls (Speechley et al., 2010; Dudley et al., 2016).

A number of studies also connect executive impairment in schizophrenia with the *severity* of positive symptoms such as delusions and hallucinations (Guillem et al., 2008; Lesh et al., 2011; Fioravanti et al., 2012). Particularly interesting in this respect are the studies focusing on EF performance in Sz-AVH subjects. A recent study that specifically compares controls and Sz-AVH patients is the one conducted by Brébion et al. (2016). In this experiment, participants completed a simple verbal memory task involving free recall and recognition of lists of words with different structures (i.e., high-frequency, low-frequency, and semantically organizable words). The experimenters recorded the number of words recalled as well as the number of extra-list intrusions and false recognitions. Notably, both intrusions and false recognitions were reliably associated with AVH, and these mistakes did not seem dependent on the semantic structure of words (p. 7). Another recent study conducted by Toh et al. (2020) employs a battery of tasks to assess the degree of EF disruption in three subgroups diagnosed with schizophrenia: current voice-hearers, past voice-hearers, and never voice-hearers. Notably, current voice-hearers were found to be more impaired in the inhibition and visual learning domain (i.e., visual organization tasks requiring elements of planning), both with respect to controls and clinical subjects who had not recently experienced AVH. The idea that Sz-AVH subjects would exhibit specific issues with the inhibition of irrelevant stimuli – as well as with WM – has also been supported by studies employing the Inhibition of Currently Irrelevant Memories task (ICIM) – see Waters et al. (2003) and Paulik et al. (2007). In these experiments participants are asked to identify a number of target pictures over four rounds, while filtering out potential distractors – i.e., target pictures that appeared in previous rounds. The ability to inhibit previously relevant items was found to be diminished both in Sz-AVH subjects (Waters et al., 2003) and in non-clinical subjects predisposed to hallucinations (Paulik et al., 2007; Gupta et al., 2018). Although more research is needed to corroborate these findings, they support a possible connection between AVH severity and poor EF performance. In particular, these results suggest that Sz-AVH subjects – along with people at-risk of developing hallucinations – may encounter specific EF difficulties involving *inhibition* and *WM*.^5^

In the remainder of the section, we draw on this evidence to substantiate our view on EF difficulties in the Sz-AVH population. We begin by exploring the broader IS profile exhibited by Sz-AVH subjects, we list some of its most disruptive features and explain how EF problems may account for them. Generally speaking, it is expected that Sz-AVH subjects will often lack control over their IS because their self-monitoring mechanisms, partially dependent on EF elements, may fail to make the required adjustments to match outputs to desired goals. Here we further detail this proposal by suggesting that many IS episodes in Sz-AVH subjects would be characterized by an *uncontrolled/uncontrollable* character. This broader feature might in turn depend on more specific IS characteristics, such as:

• Overflowing and Distracting: Sz-AVH subjects may experience more *intrusions* in IS, and this would negatively affect their performance in verbal, inhibition, shifting, and planning tasks. For instance, in the study conducted by Alderson-Day et al. (2014), vulnerability to AVH experiences correlates with the presence of other people in IS (see also de Sousa et al., 2016; Rosen et al., 2018). This characteristic may in turn be connected with a higher degree of *interference* with task completion, as the presence of other people in IS would arguably represent a major distracting factor.
• Negative, Emotionally Relevant and Dystonic: The IS episodes experienced by Sz-AVH subjects may be more negative in content, or they may be appraised more negatively (Fernyhough, 2004; Hugdahl et al., 2012). They might also be harder to ignore due to their emotional relevance (e.g., self-derogatory comments) or their dystonic character (i.e., failure to align with the person’s self-attributed thoughts and emotions) – see López-Silva (2016).
• Multiple, Fragmented: The IS episodes experienced by Sz-AVH subjects are likely to be multiple (i.e., focusing on different aspects of experience at the same time) fragmented (i.e., distributed across more than one “voice,” not temporally coordinated or synchronized), and externally attributed (i.e., appraised as coming from an external source as opposed to self-originated) – see Langland-Hassan (2008). IS experiences characterized by fragmentation would disrupt the acquisition of a stable viewpoint, as different perspectives simultaneously coexist in the subject (see Hurlburt, 2011 for a similar view on bulimia nervosa).

So far, we have presented some evidence of specific EF difficulties in the Sz-AVH population and we have offered some reasons to think that these subjects would exhibit an uncontrolled IS profile. In what follows we suggest that these underlying EF issues impact IS in Sz-AVH subjects, but are also exacerbated by the kind of IS exhibited by this population. On the one hand, the EF difficulties experienced by Sz-AVH subjects impact their use of IS, thereby contributing to its uncontrolled character. On the other hand, an uncontrolled IS is likely to have important effects on executive functioning as well. In a nutshell, underlying EF problems result in an uncontrolled IS profile, which in turn engenders additional issues in the executive domain.

The evidence discussed in the first part of the section points to global EF impairments in the Sz-AVH population that are more severe with respect to controls as well as people with schizophrenia *simpliciter*. Such global EF issues are likely to impact self-monitoring and self-control to a significant extent. Self-monitoring is a control mechanism that enables subjects to monitor and correct errors, and so to successfully achieve goals and complete actions (Pacherie, 2008). While the execution of some plans, like verbally expressing a thought or reaching for a glass, may not engage EF components, other goals require a hierarchical implementation that may fail if EF components do not work properly. Specific EF impairments such as false recognitions or intrusions in inhibition tasks (Hugdahl et al., 2012), or the inability to filter out irrelevant items in WM (Waters et al., 2003; Brébion et al., 2016) are thus likely to significantly impact self-monitoring. Disruptions in self-monitoring would result in a diminished control, which can in turn give rise to experiences of alienation in extreme cases (i.e., complete lack of control). In fact, many researchers have suggested that passivity phenomena in schizophrenia relate to failures in self-monitoring (Feinberg, 1978; Frith, 1992), and in particular that AVH are the result of failures in monitoring mechanisms applied to IS production (Frith, 1992; Langland-Hassan, 2008; Vicente, 2014). Such issues with self-monitoring at the level of implementing basic goals will also carry over to higher order plans that recruit EF components that are themselves compromised. In particular, they may affect what has been labeled “dialogic IS” (Fernyhough, 2004), which refers to the conversations we have with ourselves (see below). In this respect, the overflowing and distracting nature of IS in Sz-AVH (i.e., the impossibility to keep it focused on some goal), due to self-monitoring issues, may ultimately relate to inhibition and WM difficulties. Indeed, a subject’s inability to inhibit prepotent responses, filter out distractors, keep in mind the relevant aspects of a task, and selectively focus attention on them may generate more intrusions in IS, thereby making it more uncontrolled.

We then suggest that some further EF issues would appear as a *consequence* of such uncontrolled IS profile. That is, Sz-AVH subjects may have a harder time employing their IS effectively and channeling it toward the task at hand. For instance, going back to the features listed above, we suggest that *evaluative* and *motivational* aspects of IS are likely to have an impact on EF tasks, as a negative, emotionally relevant, and dystonic background commentary on one’s performance is likely to be particularly disruptive. Similarly, fragmentation and multiplicity in IS would make it difficult for subjects to employ IS for self-direction and self-regulation purposes, as their experience would be harder to integrate and unify under a stable point of view. IS experiences characterized by multiplicity would thus prevent subjects from focusing on the task at hand, as several aspects of reality present themselves as relevant at the same time. So, the use of IS in Sz-AVH subjects would create additional difficulties in EF tasks, and specifically in those tasks where IS is said to be helpful in the neurotypical population. If a subject experiences one’s own IS as being uncontrolled, negative, and fragmented, all the Core EF are likely to suffer: such a profile arguably makes it harder to manipulate information online (WM), to suppress irrelevant responses (inhibition), and to flexibly transition between different aspects of a given task (switching). HO-EF such as planning, reasoning, or problem-solving, usually considered to significantly engage IS, would be negatively affected too.

In the particular case of AVH, there is evidence supporting the idea that AVH experiences would negatively affect EF performance by attracting attentional focus and thereby impairing cognitive control, in particular *inhibition*. For instance, Hugdahl et al. (2012) ran a dichotic listening task on a sample of patients diagnosed with schizophrenia. Within these tasks, subjects are asked to attend to external speech sounds coming from their left and right. Given the already known Right Ear Advantage phenomenon (REA), the request to focus on the right ear is usually taken to measure attentional focus (FR, synergic action), whereas the request to focus on the left ear helps to measure control or inhibition (FL, antagonistic action). The results collected by Hugdahl et al. (2012) support the idea that Sz-AVH subjects would experience more “voice interference,” and the effect would be particularly strong given that the task employs a similar sensory modality (i.e., acoustically presented speech sound). Notably, this effect appears to correlate with AVH severity: “The more frequent the hallucinations, the less were these patients able to direct attention to the right ear syllable in the FR instruction condition, and to use cognitive control to increase reporting of the left ear syllable in the FL instruction condition” (p. 304).

To enhance self-regulation, IS has to resemble a well-structured conversation as much as possible. The Vygotskyan hypothesis is that children internalize patterns of conversation that have proven useful in regulating their behavior (e.g., directing their attention to relevant features of the environment, sequencing their behavior in a certain way, alerting them about switching, etc.). In order to be a tool for self-regulation, IS should thus resemble *that* kind of regulatory conversation. Now, imagine a caregiver playing with a child trying to solve a jigsaw puzzle who instead of telling the kid “this piece here,” “look there,” etc., starts speaking about a movie, then lunch, then how ugly the puzzle is, etc. Instead of regulating the child’s behavior, the caregiver will only be distracting the child and interfering with her performance, even more so if the caregiver’s talk is loaded with negative evaluations. Similarly, there is no reason why talking to ourselves should not be a distractor rather than a useful resource. In order to be a useful resource IS has to be itself under control. After all, speaking to oneself in silence is an action that usually forms part of a larger plan of actions; as such it has to be monitored so that it adjusts to the goals of the plan, and corrected if it does not. A controlled form of IS will be structured in the way overt conversations are structured, i.e., around a question under discussion (or QUD, see Roberts, 1996). A less controlled IS will not respect the QUD dynamics, and subjects will experience a less robust feeling of agency concerning their IS, since their self-talk will *switch* from one question to another without really addressing any of them, that is, without achieving the goal of opening a conversation. In such cases, subjects will experience their IS as being intrusive. This might be seen as the result of multiplicity and fragmentation within IS. Less controlled forms of IS may result in contents that are themselves disruptive (i.e., independently of not being part of a conversation). Instances of disruptive content would feature prominently in negative or evaluative IS experiences (e.g., self-derogatory talk). In the extreme case of AVH, IS will be misattributed and experienced as alien. If this is correct, uncontrolled IS may therefore become disruptive when it comes to EF tasks, which by definition require the subject to focus on relevant stimuli and filter out non-relevant ones.

We realize that the view we defend could raise a potential circularity worry: after all, impaired EF would be responsible for uncontrolled IS, and at the same time uncontrolled IS would cause problems in EF tasks. To clarify: our point is that some EF impairments are not *caused by* uncontrolled forms of IS but they are instead, together with self-monitoring issues, what *causes* unregulated IS. However, if IS is uncontrolled, insofar as it is usually recruited in EF tasks, it will generate further EF problems. In order for IS to be of any use (with respect to EF), subjects need to already possess a minimally functioning EF system that inhibits, updates, etc., and is eventually capable of controlling IS. Otherwise, IS *per se* is of no help, or, even worse, it can be disruptive. To sum up: we do not claim that *all* EF problems in Sz-AVH subjects stem from uncontrolled IS. Rather, the claim is that, to the extent that EF tasks tend to recruit IS, an uncontrolled and distracting IS will have a negative impact in such tasks.

## Executive Function and Inner Speech in ASC

Autistic Spectrum Condition (ASC) is a neurodevelopmental condition defined by deficits in social communication and interaction and restrictive and repetitive patterns of behavior (American Psychiatric Association [APA], 2013). In this section we first review some existing findings on how ASC subjects perform in EF tasks, and then we connect these results with our broader hypothesis concerning the use of IS in this population.

Several studies show that EF impairments are prevalent in Autistic Spectrum Conditions. Such results led to the formulation of the theory of ASC as deficits in executive functioning (Ozonoff et al., 1991; Hughes et al., 1994; Hill, 2004). The executive dysfunction theory was taken to account for many of the non-social aspects of autism and was the only theory acknowledging both the cognitive and motor aspects of ASC (Rajendran and Mitchell, 2007), compared with the Theory of Mind theory (Baron-Cohen et al., 1985) and the Weak Central Coherence theory (Frith, 1989). This theory also purported to account for theory of mind deficits within ASC, as EF difficulties would make it harder for a subject to simultaneously hold two representations in mind, i.e., one about reality and the other about the other person’s representation of reality. Overall, a recent meta-analysis of EF in ASC confirms a broad executive dysfunction (Demetriou et al., 2018), although EF deficits are not currently regarded as core symptoms of ASC (Friedman and Sterling, 2019).

Empirical studies show that evidence is mixed and EF performance in autism depends on a number of factors, including the function evaluated, the population of the studies, the subtypes of autism, etc. Regarding *working memory*, some studies found significant working memory impairments in ASC (Boucher et al., 2012; Kercood et al., 2014) and WM impairments have been reported to be related to communication and socialization deficits (Gilotty et al., 2002; Oliveras-Rentas et al., 2012) and restrictive and repetitive behaviors (Lopez et al., 2005; Sachse et al., 2013) in individuals with ASC. This general view, however, receives a more detailed treatment when studies look for differences between *verbal* and *visuospatial* working memory, the results then being that visuospatial working memory is more impaired than verbal working memory (Kercood et al., 2014; Wang et al., 2017) or even that verbal WM is intact (Williams et al., 2005; Cui et al., 2010). This is relevant to our purpose of investigating the presence and use of IS in the ASC population. Notably, in the studies conducted by Williams and colleagues, performance both among controls and ASC people in WM tasks have been found to decline substantially when IS use is obstructed through articulatory suppression (Williams et al., 2008 for evidence about children; Williams et al., 2012 for evidence about adults). This already indicates that some use of IS is in place in ASC populations and that it seems to play a central role in recalling tasks.

With respect to *inhibition*, studies with ASC people show general patterns of deficits of inhibitory control (Geurts et al., 2014), also associated with restricted and repetitive behaviors (South et al., 2007; Mosconi et al., 2009). Xiao et al. (2012) also show inhibitory dysfunction in high-functioning adolescents with ASC, using the Go/no-go and the Stroop tasks using functional imaging techniques. Similarly, Schmitt et al. (2018) show inhibitory control deficits in ASC involving failures to strategically delay behavioral response onset. In contrast with this, Lopez et al. (2005) report good performance on response inhibition (and on WM too) in adults, and Adams and Jarrold (2012) find that children with autism have no difficulty inhibiting prepotent responses in a stop-signal task.^6^

Regarding the remaining core component of EF in ASC, *task-switching*, once again the evidence appears to be mixed. Several studies have shown impairment in task-switching using the WCST (see Willcutt et al., 2008; Landry and Al-Taie, 2016). These studies found a large effect size for the difference in task-switching between ASC and typically developing (TD) groups, with ASC adults scoring particularly high in terms of perseverative errors in the card-sorting task (Lopez et al., 2005). However, Geurts et al. (2009) claim that such tasks are not related to task-switching *per se* but to the whole Core EF construct. To rule out the competing explanations for failure in the WCST, some researchers employ the Intra Dimensional-Extra Dimensional shift task (ID/ED). This test is similar to the WCST but requires participants to shift both intra-dimensionally (e.g., between different colors) and extra-dimensionally (e.g., from color to shape). Evidence is mixed here, again, as Ozonoff et al. (2004) reported difficulties with ED-shifts but not with ID-shifts in the largest autism study to date on children (but see Landa, 2007, for contrary evidence). Geurts et al. (2009) argue that attention deficits can also be responsible for failures in ED-shifts, *combined with* the fact that participants that do not reach the final stages of the ID/ED task are automatically given the highest error score. This seems to be particularly relevant given how the task is designed, with the most difficult parts – i.e., extra-dimensional shifts – always appearing at the end. As a consequence, subjects who have a hard time staying focused (no matter the reason) would inevitably score poorly. Attention deficits have been argued to be central in autism (Allen and Courchesne, 2001), although once again the high comorbidity with ADHD may be a confounding factor (see section “Further Considerations”).

Interestingly, it has been shown that ASC children show limited use of IS in task-switching paradigms (Whitehouse et al., 2006) and arithmetic task-switching tasks (Holland and Low, 2010), and this negatively impacts their performance. Actually, Geurts et al. (2009) report that the task-switching performance of ASC adults improves if they are informed that the rule has changed. That is, ASC people can perform as well as their TD peers in pure switching tasks (e.g., just switching from one way of sorting cards to another) once they are *explicitly told* that the task is different. This suggests that TD peers, who do not need such an explicit reminder, may be alerting themselves about changes in rules using their own IS (see section “IS in ASC and Sz-AVH: How It Relates to EF” below).

Finally, experimental evidence of *planning* and *problem-solving* abilities in ASC people point to the fact that they encounter planning difficulties (e.g., Lopez et al., 2005 for results on adults and Wallace et al., 2009 for results on adolescents). They have trouble organizing their daily life, keeping up with (social) activities or coping with unregulated stretches of time (Ozonoff et al., 2002; American Psychiatric Association [APA], 2013). A recent quantitative meta-review (Dubbelink and Geurts, 2017) concludes that people with ASC show poorer planning performance than TD individuals (moderate difference in size) that is consistent across lifespan (age), various types of planning tasks, and intellectual abilities.^7^ Other studies examining the use of IS in ASC with respect to planning and problem-solving showed that children failed to use IS in planning movement compared to visuospatial resources (Holland and Low, 2010), and that planning was not verbally mediated in adults (Williams et al., 2012).

Drawing on these experimental results, we suggest that ASC people may not capitalize on the enhancing potential that an appropriate use of IS might have for planning and problem-solving. However, they may perform well in certain planning tasks that can be solved using visuospatial resources. Geurts et al. (2009) show that inflexible everyday behavior in ASC fails to be adequately reflected by experimental results in tasks measuring flexibility (such as the WCST). In other words, the clinical evidence about behavioral flexibility conflicts with the experimental evidence about cognitive flexibility. Something similar may apply to planning: performance in tasks such as the Tower of London may be poor predictors of coping with real-life planning demands, especially if such tasks allow for the use of compensatory strategies such as visuospatial planning that may be more difficult to implement in real-life situations. The empirical evidence we reviewed about EF difficulties and IS use in ASC suggest that ASC people have a *diminished* use of IS compared to neurotypicals (see also Mulvihill et al., 2020 for a recent review of self-directed speech –including IS – in children with ASC, ADHD, and DLD). On the one hand, they exhibit good performance in verbal WM, which is, on the face of it, an instance of IS recruitment. But on the other hand, studies on other core EF components such as inhibition or cognitive flexibility, as well as higher-order ones such as planning and problem-solving, suggest that an insufficient recruitment of IS may be responsible for their weak performance in such domains. The idea that ASC children would have trouble engaging in self-instruction, and thereby in completing EF tasks, is not entirely new (see Russell et al., 1999). This hypothesis is also congenial to Fernyhough’s suggestion (1996; also mentioned in Williams et al., 2012) that ASC people may have a monologic rather than dialogic form of IS. This would not imply a complete inability to produce IS, like the one exhibited by some people with aphasia who are unable to judge whether two words rhyme (Geva et al., 2011). What we suggest is rather that people with ASC would not appropriately employ IS in the service of self-regulation and/or thinking, that is in processes in which IS typically takes the form of a conversation where proper speech acts are produced (i.e., assertions, questions, commands, etc.). This would imply that at least some EF issues in ASC are related to a diminished recruitment or lack of use of IS.

It should be noted that ‘diminished use (or lack) of IS’ refers to a form of IS that characterizes it as precisely a form of proper speech, in analogy with outer speech. There is a difference in kind between simply holding verbal information in mind (as in WM) and engaging in self-talk for thinking and other cognitive functions. The process of holding in mind verbal information does not require to produce meaningful IS and not even to perform proper speech acts. For instance, unlike typical speech production, the former process does not begin with a “message” to be expressed. Therefore, the crucial difference here does not rest on the relevant process being dialogic versus monologic (as Fernyhough, 2004 has suggested), but rather on the fact that the self-talk for thinking is an instance of *proper speech* while holding information in mind is not. The kind of IS that relates to effective EF in neurotypicals is what we could call “proper IS” (Martínez-Manrique and Vicente, 2015): people give themselves verbal instructions, focus their attention by using verbal commands, assertions or reminders, motivate themselves using exclamatives, etc. In general, neurotypicals use a kind of IS in the service of self-regulation that resembles their overt speech (Martínez-Manrique and Vicente, 2010; Jorba and Vicente, 2014). The *kind of IS* that is quite consistently found lacking or diminished in ASC (Williams et al., 2008, Williams et al., 2012) is precisely this proper form of IS (see also Hurlburt et al., 1994).

The claim that lacking or diminished use of IS might be responsible for EF problems encounters some exceptions, which also end up supporting the claim. Williams et al. (2012), point out that not recruiting IS for EF tasks (but also, for other functions) may not be a general feature of ASC people: in their study, adults diagnosed with ASC who had comparatively better socio-communicative performance showed a decline in performance for planning tasks under verbal interference. As Alderson-Day and Fernyhough (2015) point out: “Communication scores for ASD participants on the Autism Diagnostic Observation Schedule (ADOS; Lord et al., 2000) and Autism Quotient (Baron-Cohen et al., 2001) were observed to predict articulatory suppression effects during *planning*, suggesting a link between communicative ability and lack of IS specifically to support *problem-solving*” (p. 946, emphasis ours). The DSM-5 distinguishes two dimensions in ASC: socio-communicative problems, and restrictive and repetitive patterns of behavior. Similarly, the ADOS diagnostic test has separate scores for the socio-affective dimension, and stereotypical and repetitive behavior and fixed interests. Probably, the fewer socio-communicative problems a person has, the more typically verbal in general she will be (except if she has further problems with language). And actually, ASC people with lower scores (that is, fewer problems) in the socio-affective dimension of the ADOS are overall more “conversational”: they keep track of the common ground (e.g., they introduce new referents with indefinites and not with definites or proper names); they address the question under discussion (e.g., they observe the principle of relevance): and they use tags and other discourse particles that engage their interlocutor (e.g., tags like “right?”, beginnings such as “now, look!”, expressions like “sweetie,” etc.): see Castroviejo and Vicente (unpublished).^8^

So it is likely that people who have a more fluent “proper” conversation will also recruit IS more often, and for the same functions for which most overt speech is used (as happens in the neurotypical population). Then the hypothesis is that *only* ASC people who have fewer problems in maintaining a conversation (and who, correspondingly, had fewer problems in learning how to converse) will make a more or less typical use of IS. In contrast, people who have more socio-communicative problems will also exhibit more problems in having fluent conversations as well as in recruiting IS in those situations where the aid of overt speech would also be helpful. In such cases, the increased propensity for visual over verbal encoding may reflect a compensatory response to reduced verbal ability and self-directed talk regulatory effectiveness (Lidstone et al., 2009; Russell-Smith et al., 2014).

## Is in ASC and Sz-AVH: How It Relates to EF

In the previous sections we have reviewed evidence concerning how IS is supposed to be involved in EF tasks, and we have offered some suggestions on the kinds of IS profiles exhibited by people diagnosed with ASC and by Sz-AVH people. In this section we aim at bringing together these results to offer a more coherent picture of how different uses of IS may impact EF in these two populations as well as in neurotypicals.

Given the evidence discussed above, IS appears to play an important role in EF tasks. It may be questioned whether IS plays the same role across different populations: for example, we have seen that ASC people can perform well on planning tasks such as the Tower of London without the aid of IS, while neurotypicals perform worse on average if unable to use IS. This means that the use of IS is not crucial in some EF tasks across subjects (though it may be crucial for a subset of them). However, overall, it can be maintained that IS enhances performance in EF tasks, even in the “classical” experimental tasks. In Go/No-go tasks, for instance, it helps to label the stimuli in IS (Alderson-Day and Fernyhough, 2015). As Lupyan and Bergen (2016) have stressed:

“For example, labeling one’s actions supports the integration of event representations (Karbach et al., 2011), overt self-directed speech can improve performance on such tasks as visual search by helping to activate visual properties of the targets (Lupyan and Swingley, 2012). Conversely, interfering with (covert) verbalization impairs the ability to flexibly switch from one task to another (Baddeley et al., 2001; Emerson and Miyake, 2003), hinting that normal task-switching performance is aided by such self-directed, covert instruction.” (p. 410).

But even in those tasks where the use of IS has not been directly tested, it can be argued that the recruitment of IS improves performance. A case in point is, again, task-switching. As noted above, Geurts et al. (2009) report that the performance of ASC subjects in the WCST improves when they are informed that the rule has changed. This is presumably so because by telling ourselves that the rule has changed, or which is the new rule, this information is thereby made conscious, that is, we become aware of something we were not aware of before and so we can act upon it. There is also some evidence that communicative acts like this also help neurotypicals in very different tasks, from reorienting themselves (Shusterman et al., 2011) to encoding spatial relationships (Landau et al., 2010).

Spelke’s reorientation data were widely discussed in the early 2000s: after being disoriented in a room with three white walls and a fourth red wall of different sizes, subjects experienced reorientation problems under verbal interference. Shusterman et al. (2011) later observed that reorientation was significantly improved if 4-year-olds, who had the same reorientation problems as adults under verbal interference, were told at the beginning of the test that the object they had to look for (i.e., a sticker) was “hidden at the red/white wall.” Non-spatial but task-relevant expressions – such as “The red/white wall can help you get the sticker” – also enhanced children’s search. A likely reading of these results is that adults have to be able to verbally remind themselves where the hidden object is, something they cannot accomplish as effectively under verbal interference. Similarly, in Shusterman et al. (2011) study children’s performance improves exactly when these verbal reminders are made more explicit. In other words, orientation is improved if subjects are able to use IS. Landau et al. (2010), on the other hand, presented findings on how language can enrich how we represent a scene (so that we can later on recall more details of it). Their results are best interpreted if we think about language as a means of directing attention, as the linguistic input helps hearers focus their attention on aspects of the scene that they would have not otherwise encoded. For instance, we may watch a square divided in two halves by a vertical line, the right part green, the left part red. When, after a brief exposure, we have to pick out the square from a set of four pictures with different distributions of green and red, we have real problems to retrieve the correct distribution of colors. We know the colors are green and red, but do not know where they go: we do not bind colors to the left and right parts of the square. Landau et al. (2010) tested whether such binding could be improved through language. They tested 4-year-olds in several conditions: experimenters labeled the whole square (“this is a dax”), or they said “the red is touching the green,” or they drew the children’s attention to the red part (“look at the red!”), or, finally, they directly told them: “the red is on the left.” Children improved over the no-stimulus condition only in the last of these conditions. According to Landau et al. (2010), once children’s attention is directed toward the relation between the two colors, they are able to solve the binding problem. Again, a case can be made for thinking that adults who succeed in these tasks do so because they are telling themselves what the experimenter says aloud to the 4-year-olds.

Notably, these are not issues about preferences in task performance, i.e., whether some subjects prefer to solve the task using language while others prefer imagistic thinking. It is rather an issue about the intrinsic limitations in terms of attentional resources that visualizations may have as opposed to linguistic encodings. In other tasks the use of IS may even be detrimental, as showcased by the “verbal overshadowing effect” (Chin and Schooler, 2008).^9^ But, generally speaking, IS simplifies EF tasks and so solving such tasks without IS will at least increase one’s cognitive load. The point made by Geurts et al. (2009) about the improvement observed in the WCST after communicating that the rule had changed seems to tell us something similar. In these cases, there may be problems attending to failed responses properly, or realizing that one has made enough mistakes before trying another rule, or there might be problems inhibiting a preponderant response. Whatever the reason for not switching in time, being told “this is a new rule” obviously reduces cognitive load. It simplifies the task in a similar way as being told “the red is on the left” simplifies Landau et al. (2010)’s task. If subjects tell themselves things such as “same rule,” or “new rule,” at each trial, they will be able to simplify the task considerably. For instance, hearing themselves say “same rule” after a mistake will alert them about an inconsistency that prompts them to look for a new rule. Such linguistic encoding of the input increases vigilance at very low cost. A similar process has been detected by researchers studying task-switching in subjects with schizophrenia. Indeed, some studies suggest that participants with schizophrenia improve their performance in EF tasks when they are explicitly asked to verbalize their thought processes. For instance, Perry et al. (2001) ran the WCST with a group of schizophrenic subjects, and reported that their performance significantly improved when they were asked to verbalize their current sorting category for each card. These results suggest that verbalizing (either overtly or covertly) might be an important component of successful performance even on seemingly non-verbal tasks such as the WCST.

Verbalization, be it overt or inner, thus improves EF performance in probably all the EF-subcomponents, which means that subjects who would normally fail to recruit IS altogether (ASC) as well as subjects who lack control over their IS (Sz-AVH) face clear EF disadvantages. In terms of intervention, both kinds of populations could be encouraged to mobilize IS as a tool in service of these tasks. On the one hand, people with ASC benefit from more explicit instructions - such as being told that the rule has changed – because it facilitates holding and manipulating information in WM, and reduces cognitive load with respect to switching and planning. Intervention in ASC should therefore strive at making subjects more likely to use IS. On the other hand, Sz-AVH people benefit from a guided and explicit verbalization because it filters out possible distractors and keeps people focused on the task at hand. Intervention in this case should aim at keeping IS under control, so that subjects themselves are able to channel it toward the task at stake.

At the same time it is important to stress the alternative strategies used by ASC people to bypass the failure to recruit IS. ASC people are able to compensate for a diminished use of IS in EF tasks by using visuospatial formats, and this seems to be a strength in the ASC population across the board (Morsanyi et al., 2019). However, according to the literature reviewed above, visuospatial formats also have intrinsic limits. Without having to endorse views such as Bermudez’s (2003) or Gauker’s (2011), which deny that imagistic formats are able to be the basis of propositional thinking, it seems to be the case that language considerably simplifies EF tasks. If this is correct, compensatory mechanisms will be far from perfect substitutes to the use of language, but they would still be able to compensate to an extent. However, with respect to AVH and similar profiles characterized by uncontrolled and intrusive IS, intervention on IS seems more pressing, given the lack of any compensatory mechanism. In other words: if you have an overly active IS, your performance in EF tasks will inevitably decline because of interference and distraction, that is, unless you are able to gain control over it. However, if you do not use IS for these tasks, you have more wiggle room to employ alternative strategies, e.g., visual or spatial, and thus perform better.

We have mainly discussed ASC and Sz-AVH because they illustrate two different ways in which issues about IS can affect EF. This comparison should not overlook the fact that these two conditions have a different relationship to IS overall. Whereas Sz-AVH is mainly described as an IS *disturbance*, the diminished role of IS in ASC is *not key* to ASC as a condition *per se*, but probably originates from socio-communicative developmental trajectories. However, the more substantial difference between the two conditions is the following: in Sz-AVH IS is (too) active and uncontrolled due to pre-existing EF issues, whereas in ASC IS does not appear as active as in the TD population and this leads to diminished EF performance. We have suggested that these differences in EF profiles are due to the IS profiles that each population presents. An uncontrolled form of IS in Sz-AVH leads to important EF issues, and diminished use or lack of use of IS in ASC possibly leads to EF difficulties in ASC. Overall, we think that the focus on these two populations and the hypothesis advanced shed some light on both conditions and more generally on the role played by IS in clinical and non-clinical populations, thus paving the way for further exploration.

## Further Considerations

There are a few issues that we should flag before we conclude. One important limitation in our discussion of ASC concerns the fact that potentially confounding factors, such as IQ or verbal competency, are not ruled out by many of the studies we examine. To address such important worries, future studies investigating the role of IS in ASC EF-profiles should carefully control for these variables. The same is true about the high comorbidity between ASC and ADHD, given that most studies on ASC do not directly control for it (but see Sinzig et al., 2008).^10^ As a result, in many cases it is difficult to establish whether EF difficulties are ASC-specific, or whether they actually result from the simultaneous presence of ADHD in a majority of subjects.

Our hypothesis would probably be enriched by analyzing the role of IS specifically in ADHD. Although it is a less researched area, there is some evidence pointing toward *dysfunctions* in self-regulation and *delay* in the internalization from overt to covert speech, with potentially important implications on EF. For instance, Barkley (1997) suggests that a delay of self-directed speech (SDS) internalization negatively impacts its guiding function that he describes as fundamental to self-control, problem solving, moral reasoning, and metacognition. More recent studies support the idea of a delayed progression from overt to internalized SDS, as well as the preponderance of task-irrelevant content. Although such delay could be responsible for some EF dysfunctions, the evidence is still far from conclusive and the interpretations regarding the self-regulatory efficacy of SDS in ADHD are somewhat speculative (Mulvihill et al., 2020). That said, some encouraging results may be gleaned from some successful academic interventions on students with ADHD. As reported by Miranda et al. (2009), one of the cognitive-behavioral techniques that has shown the greatest efficacy employs self-instruction (i.e., self-talk) to foster sequential thinking, facilitate the comprehension of situations, spontaneously generate strategies and mediators, and use these mediators to guide and control behavior (Meichenbaum and Goodman, 1971). Other successful programs, such as “Thinking aloud” (Bash and Camp, 1980) and “Stop and think” (Kendall et al., 1980), similarly encourage the use of self-instructional techniques to support problem-solving and contingency management.

It is likely that the delay in the use of IS in ADHD relates to lack of attention in regulatory practices. If the child fails to be regulated by the overt speech of her caregivers, she will also fail to internalize this kind of speech efficiently. On the other hand, it is also probable that, like in Sz-AVH, IS is not adequately controlled in ADHD, thereby becoming more a distractor than an aid. Thus, the abovementioned interventions can be seen as aiming in good part at structuring subjects’ self-talk. If this is correct, the IS profile in ADHD would be significantly different from the ASC profile. In ADHD, there would be core EF deficits that affect their use of IS, resulting in an inefficient IS, i.e., an IS that cannot be used for improving EF. By contrast, in ASC there would be no such deficits, as the attested deficits in EF would simply be the result of not recruiting IS often enough, i.e., not recruiting a tool that can enhance EF.

Another confounding factor in studies about IS in ASC is language delay and *ASC* + *DLD* (*developmental language disorders*) *comorbidity*. In Section “Executive Function and Inner Speech in ASC,” we have discussed studies that prima facie show a diminished use of IS in EF tasks in the ASC profile, such as the one conducted by Williams et al. (2012). Other studies and pieces of evidence report a generally diminished use of IS in ASC. For example, Hurlburt et al. (1994), using Hurlburt’s Descriptive Sampling Method (DES), examined inner experiences in the daily life of three Asperger people, finding no use of IS. Also, first-person accounts such as, famously, Temple Grandin’s memoir (Grandin, 1995) have made popular the idea that Asperger people may not recruit IS at all. However, not all the results showing diminished use of IS in ASC are equally compelling, for they precisely investigate the ASC + DLD profile without controlling for such comorbidity.

Language acquisition delay is widespread in ASC (although not as much in the Asperger profile), and there is a growing consensus that ASC + DLD comorbidity is indeed quite frequent (Tager-Flusberg, 2000). Studies that work with ASC people who have some deficits in structural language, and which show that such people do not recruit IS as often as neurotypicals, do not show that IS is differently recruited in the ASC profile as such. It is predictable that people who have some kind of problem or delay with language competence more generally will have issues with IS. For instance, a study by Campbell et al. (2017) reports a lack of recruitment of IS in ASC in task-switching tasks. They suggest that visual abilities may play a unique role in the ASC population as they seem to capitalize on them given their “weak verbal abilities.” However, these results are not illustrative about the use of IS in ASC. Studies on DLD also connect delayed language development with scarcer IS presence. Overall, it seems that children with DLD use self-directed talk to a lesser extent than TD peers, with a negative impact on tasks such as the Tower of London (Lidstone et al., 2012). Therefore, it is likely that the above results have more to do with linguistic problems than with the autistic condition.

Reviewing studies on DLD, IS, and EF is beyond the scope of this paper, although it is predictable that children with DLD will also show a diminished use of IS. A point for further exploration concerns the role of IS in EF tasks in DLD, ADHD, as well as in other conditions such as depression, anxiety, and obsessive-compulsive disorders, where there is already research on IS behavior (Harrington and Blankenship, 2002; Nolen-Hoeksema et al., 2004; Perrone-Bertolotti et al., 2014; Nalborczyk et al., 2017). Besides shedding some light on how different IS profiles may impact EF in the ASC and AVH populations, we hope that the dimensions of IS that we have uncovered would prove helpful in investigating these and other clinical conditions.

## Author Contributions

All authors listed have made a substantial, direct and intellectual contribution to the work, and approved it for publication.

## Conflict of Interest

The authors declare that the research was conducted in the absence of any commercial or financial relationships that could be construed as a potential conflict of interest.

## Footnotes

1 In line with the autism rights movement and neurodiversity movement more generally, we use the label ‘Autistic Spectrum *Conditions’* (ASC) instead of ‘Autistic Spectrum *Disorder’* (ASD) in this paper. However, we acknowledge that ASD is still widely used as a diagnostic label (following the DSM-5 classification), and that most of the studies discussed here employ this terminology.

2 The particular constructs underlying EF tend to be labeled differently by different research groups, thereby enhancing terminological confusion (e.g., attentional control, effortful control, self-regulation, self-monitoring; see Snyder et al., 2015).

3 Based on studies on the activation of the mouth motor system when learning novel categories and real abstract words in adults, and developmental studies on the interference produced by the use of pacifier in children, as well as whether gum-chewing affects adults’ online processing of abstract concepts, Borghi et al. (2018) hypothesize that IS contributes “either to the re-enactment of the acquisition experience, or the re-explanation to oneself of its meaning and/or to the information request on the conceptual meaning to others” (p. 6).

4 AVH additionally feature as a significant symptom of other psychiatric disturbances such as bipolar disorder and major depression (see Toh et al., 2015 for a review). The sole experience of AVH under stressful circumstances, especially when combined with a negative appraisal, has the potential of giving rise to clinically relevant manifestations (Fernyhough, 2004).

5 More specific proposals on the aspects of inhibition involved in AVH have been advanced by Paulik et al. (2008) and Alderson-Day et al. (2019). Both groups propose *intentional* cognitive inhibition (i.e., the ability to willfully control thoughts and memories) as the relevant mechanism in AVH development. Although their proposal deserves further theoretical and empirical scrutiny, we settle for a more general claim here, namely that Sz-AVH subjects exhibit EF impairments in the inhibition and WM domains.

6 However, they show significant inhibitory impairment on so-called “flanker” tasks, which require the identification of a target stimulus surrounded by various distracting stimuli (Adams and Jarrold, 2012, p. 1,053). An additional complication in inhibition studies in the ASC population is that in many cases the comorbidity with ADHD might be responsible for the inhibition impairments (see section “Further Considerations” below).

7 The tests used in the various studies include the Tower of London (Lopez et al., 2005), the Vygotsky Blocks Test (Wallace et al., 2009), and the tasks discussed in the meta-analysis by Dubbelink and Geurts (2017) include the Tower of Hanoi, Tower of London, Tower of California, Zoo Map test, Key search test, Planning drawing task, Truck loading, Stockings of Cambridge, Mazes, and Cognitive Assessment treatment-planning.

8 Stronger socio-affective abilities have also been found to correlate with irony comprehension (Spotorno and Noveck, 2014).

9 Verbal overshadowing occurs when verbalizing mental contents deteriorates the performance of a task in which those contents appear to be involved. For instance, in a classical experimental setting, all subjects watch a video about a certain salient individual that they will have to identify afterward. After watching the film and before testing their identification capacity, some subjects had to verbally describe the target individual while others had to read an unrelated text for the same amount of time. The results show that the subsequent performance in recognizing the individual (e.g., picking him/her out of a line-up) was poorer for those subjects that had been asked to describe the individual. The phenomenon is robust in the domain of face recognition – where it was originally demonstrated– but Chin and Schooler (2008) report that it has been observed also in decision making, problem solving, analogical reasoning, and visual imagery.

10 In this study, ASC children with ADHD comorbidity show difficulties with a battery of EF tasks, whereas ASC children without such comorbidity do not. However, other studies failed to find such differences (see Corbett et al., 2009).
